# SERS Biosensor Based on Engineered 2D-Aperiodic Nanostructure for In-Situ Detection of Viable *Brucella* Bacterium in Complex Matrix

**DOI:** 10.3390/nano11040886

**Published:** 2021-03-31

**Authors:** Massimo Rippa, Riccardo Castagna, Domenico Sagnelli, Ambra Vestri, Giorgia Borriello, Giovanna Fusco, Jun Zhou, Lucia Petti

**Affiliations:** 1 Institute of Applied Sciences and Intelligent Systems “E. Caianiello” of CNR, 80078 Pozzuoli, Italy; massimo.rippa@isasi.cnr.it (M.R.); riccardo.castagna@isasi.cnr.it (R.C.); domenico.sagnelli@isasi.cnr.it (D.S.); giovanna.fusco@izsmportici.it (G.F.); 2 Istituto Zooprofilattico Sperimentale del Mezzogiorno (IZSM), 80055 Portici, Italy; giorgia.borriello@cert.izsmportici.it; 3 Institute of Photonics, Faculty of Science, Ningbo University, Ningbo 315211, China; zhoujun@nbu.edu.cn

**Keywords:** quasi-crystals, nano-biosensing, SERS, nanocavities, bacteria

## Abstract

*Brucella* is a foodborne pathogen globally affecting both the economy and healthcare. Surface Enhanced Raman Spectroscopy (SERS) nano-biosensing can be a promising strategy for its detection. We combined high-performance quasi-crystal patterned nanocavities for Raman enhancement with the use of covalently immobilized Tbilisi bacteriophages as high-performing bio-receptors. We coupled our efficient SERS nano-biosensor to a Raman system to develop an on-field phage-based bio-sensing platform capable of monitoring the target bacteria. The developed biosensor allowed us to identify *Brucella abortus* in milk by our portable SERS device. Upon bacterial capture from samples (10^4^ cells), a signal related to the pathogen recognition was observed, proving the concrete applicability of our system for on-site and in-food detection.

The contamination of food and drinks with pathogenic bacteria is a problem affecting both the economy and healthcare. *Brucella abortus* is a central example of this problem because it is among the main zoonosis affecting the global economy [1]. It infects livestock and wild-life animals, resulting in loss of reproductive efficiency and abortion [2]; moreover, it is transmitted to humans by ingestion of contaminated food.

The gold standard method for the diagnosis of *Brucella* is blood culture, but this method is characterized by different drawbacks [3], not last the long time required for cell cultivation. Serological tests, including the agglutination test to detect the anti-lipopolysaccharide (LPS) O-antigen antibodies, are also available but are characterized by poor specificity due to structural similarities among different bacteria [4,5].

The control of Brucellosis is of prime importance in the endemic areas of poor countries, and the salubrity of animal source foods, in particular milk, is crucial to avoid the transmission of this disease from livestock to man [6]. The availability of simple and affordable tests to detect the pathogen presence in food of animal origin can therefore represent an important instrument to limit human outbreaks. Efficient analyses for the detection of *Brucella* are already available but not without important compromises: They are usually time-consuming, costly, and unsuitable for the on-site pathogen detection [2,7,8,9]. High-sensitivity polymerase chain reaction (PCR)-based and enzyme-linked immunosorbent assay (ELISA) methods are important examples of culture-free methods, but despite their great specificity and sensitivity, they are time-consuming and still suffering from operational simplicity [8,9]. Electrochemical Impedance Spectroscopy (EIS) is another method that has been recently employed for the *Brucella melitensis* [10] detection; however, EIS often demonstrates insufficient selectivity [11]. New attempts are therefore ongoing to develop a reliable, fast, and on-site method for *Brucella* detection [12,13].

A very promising approach to address this problem is the use of optical biosensors. Over the last decade, the combined efforts of the scientific community have driven the technological advancement towards high-performing, versatile, and compact optical devices [14] for industrial and point-of-care applications [15,16,17].

Among the different spectroscopy strategies employed to develop high-performance biosensing, Surface Enhanced Raman Spectroscopy (SERS) certainly offers appealing advantages. SERS is a label-free transduction method that exploits the plasmonic properties of metal nanomaterials (Localized Surface Plasmon Resonance, LSPR) to enhance the scattered Raman signal of several magnitudes [18] with reproducible and reliable features [13,17,18,19,20,21]. The possibility to define a SERS fingerprint spectrum for the specific identification of an analyte, from small molecules up to whole cells, is surely remarkable [22,23]. The SERS sensitivity and specificity can therefore allow a short time and effective detection of the pathogen, bypassing the need for cultures or multistep procedures.

A limiting factor of SERS-based sensors is the intrinsic loss of specificity in complex matrices that hampers their applicability and commercial distribution. The most promising strategy used in the literature to overcome this problem is the creation of a receptor layer specific for the analyte of interest. Antibodies are commonly used as receptors of biosensors, even suffering from natural sensitivity to variable operating conditions and being very expensive. A novel and workable alternative to antibodies is the use of bacteriophage viruses able to bind the organism in study. Bacteriophages not only present an excellent specificity for the host bacteria, but they are also characterized by a remarkable tolerance against critical conditions (e.g., organic solvent, extreme temperatures) [24]. Phages are viruses that can specifically infect the target host bacteria and utilize its replicative machinery to produce the progeny phages. The specific recognition and binding to the host bacteria occurs via phage tail fibres and baseplate [25]. The specificity of this phage-bacteria bond can be also higher than antibodies and other bioreceptors commonly used to detect pathogens such as aptamers and antimicrobial peptides. Moreover, phages are characterized by lower costs and can be cultured in adequately equipped microbiological laboratories. In particular, the Tbilisi bacteriophage specifically recognizes *B. abortus* and it has been used for decades for *Brucella* species identification in the diagnosis and epidemiology of brucellosis [26]. 

We already successfully showed the feasibility of our SERS nanosensors for *Brucella* analysis [12] and the quality of the achieved results encouraged us to further explore the efficiency of our technology for the detection of the live pathogen in real food samples. 

In this communication, we explored a new optimized approach in which a sensitive deterministic aperiodic nanocavity (DANC) patterned gold layer was covalently functionalized with Tbilisi (Tb) bacteriophages via diazo-coupling for a detection of the *Brucella abortus* in a food matrix. The developed sensor was also used to detect the viable form of *Brucella abortus* in milk by a portable SERS device of our creation [12] (Figure 1).

In recent times, different SERS sensors have been developed using metallic nanopatterned surfaces to reveal weak Raman scattering bio-specimen in low concentration ranges [27,28]. These nanomaterials are distinguished not only by the noble metal used but also by the pattern features. Indeed, the structure-dependent SERS enhancement is linked to the size, geometry, symmetry, and order of the nanopatterned structure.

In particular, quasi-crystal Au nanocavities (NCs) arranged in Thue-Morse array (ThMo) were chosen for the realisation of the SERS-based sensor. ThMo aperiodic geometry is generated by the iterative substitution rule: A → AB, B → BA that can be extended to two dimensions [29]. Optical properties of the ThMo nanopattern have been widely investigated and their peculiarities (singular continuous Fourier/Diffraction spectra, self-similar hierarchy of pseudoband-gap regions, omnidirectional reflectivity, and light emission enhancement) make such geometries attractive candidates for the realization of high-performance plasmonic nanosensors [23,30,31,32,33,34,35,36]. Despite the greater difficulty of both design and fabrication that they require compared to conventional periodical pattern, aperiodic arrangements show important advantages for the realization of sensing systems. As reported in the literature, they provide the necessary balance between their resonant modes and the spatial distribution of large field intensity over extended sensing areas, resulting in largely improved sensitivity respect to periodic crystals cavities, which are limited by the small overlap of the analyte with localized field [37]. Due to the higher structural disorder, the aperiodic arrays are strongly coupled in both the plasmonic near field regime (short-range coupling) and the photonic diffractive one (long-range coupling), resulting in strong in-plane multiple light scattering [38]. This enhanced scattering enables both field states that are spatially distributed over larger array areas and much longer photon dwelling times with the sensing layer compared to periodic plasmonic structures in which scattered photons easily (and faster) escape from the substrate. These characteristics improve the light–analyte interaction, enhancing the sensitivity of the system and making these types of patterns promising to develop advanced sensing devices. 

The procedure followed for the nanofabrication was previously described in the literature [39] and used here with some modifications detailed below. The plasmonic metastructures with squar-shaped NCs based on a 10th-order ThMo array were fabricated by a high-resolution electron beam lithography (EBL) system (Raith 150 EBL system by Raith GmbH, Dortmund, Germany), using ZEP 520A (Marubeni Europe plc, London, UK) as positive resist (100 nm layer). The resist was spin-coated on a 15 nm conductive ITO coated glass substrate, baked at 170 °C for 5 min, and exposed to a 10.2 pA electron beam with an area dose of 27 μC/cm^2^. After the development [40], a 50 nm gold layer was evaporated on the ZEP surface by e-beam process (SISTEC CL-400C e-beam evaporator by SISTEC, Milan, Italy). The produced quasi-crystal pattern was characterized by square NCs having a minimum distance of a = 50 nm, and a side size of d = 185 nm [23,39] with increasing edge-to-edge distances from 25 to 100 nm in a two-layer (ZEP/Au) configuration.

After the realisation of the metastructures (Figure 2), the sensors were morphologically characterized using scanning electron microscopy (SEM) (Figure 3). The analysis of micro-pictures allowed us to confirm the conformity in shape and size of the NCs produced.

The sensor surface was then functionalized with the phages. Tb phages (LGC Standards) were propagated on *Brucella abortus* in a Bio-Safety Level 3 (BSL−3) facility, using a standard protocol [12]. In particular, Tb enumeration and propagation were carried out by the double layer agar method [41].

In order to immobilize the phages, a 4-aminothiophenol (4-ATP) self-assembled monolayer (SAM) was formed on the surface for the covalent binding of Tb via diazo-coupling, as previously reported [28]. Briefly, the diazo-coupling reaction was carried out with the support of an optical microscope in order to prevent damages to the nanostructures. After the complete covering of the gold nanosurface with micrograins of sodium nitrite, acidic acid was dropped on the sodium nitrite with a consequent production of nitrous acid (HNO_2_) in the gaseous phase (bubble formation became visible on the chip surface). At last, a 10^6^ pfu/mL bacteriophage solution was added to the nanosurface and left in incubation overnight at room temperature. Several ddH_2_O washings were performed prior to air blow the chip and record the SERS spectra.

Before the detection of the alive *Brucella*, an aqueous suspension of the bacterium inactivated via formaldehyde treatment was used to obtain a reference spectrum. Precisely, 300 μL of *Brucella abortus* suspension (10^5^ CFU/mL) in water was dropped on the phage-functionalized nanostructures and they were left to react for 40 min. The sensor surface was rinsed with ddH_2_O before SERS measurement so as to remove non-captured bacteria. SERS analysis was performed by coupling a Raman system (QE Pro-Raman system by Ocean Optics, Duiven, The Netherlands) with an upright microscope Olympus BX51 (Olympus, Southend-on-Sea, England) in a backscattering configuration (Figure 1), and the spectra were collected in the range 400−2000 cm^−1^ (10 s acquisition time, 50× microscope objective with N.A. 0.75 and a laser power of 12 mW) [12]. Mean spectra were calculated from repeated measurements on different points of the sensor and on its different replicas.

Afterwards, we worked in a BSL−3 laboratory with alive *Brucella* cells suspended both in water and in milk (10^5^ CFU/mL). In particular, the contaminated sample (300 μL) was incubated on the sensor for 40 min and then washed away with ddH_2_O. The spectra were recorded on site (in the BSL−3 facility) using an optimized homemade portable Raman prototype [12].

In order to obtain an optical sensor suitable for the pathogen detection, we chose to exploit as metastructure the ThMo distribution, whose potential for sensing was already demonstrated [23,36,39]. In our previous work, we studied the plasmonic properties of ThMo-arranged NCs by Finite Difference in Time Domain (FDTD) simulations suggesting a characteristic near-field with a high spatial density of hot-spots [31]. Moreover, we demonstrated that a sensor patterned with such geometry and functionalized with 4-ATP significantly enhanced the whole spectrum of a model protein (Bovine Serum Albumin—BSA) [39]. 4-ATP is an ideal solution for the functionalisation of SERS sensors, as it is indeed an aromatic thiol able to generate well-characterized Raman bands. 4-ATP can bind the gold surface with its -SH group, forming a SAM, while the primary amine can be exploited to covalently bind receptor-like bio-molecules [42].

The EF of the fabricated ThMo SERS sensor functionalized with 4-mercaptobenzoic acid (4-MBA) was calculated to evaluate the performance of the metastructure as reported in the literature [12]

*EF* = (*I_SERS_* × *N_REF_*)/(*I_REF_* × *N_SERS_*), (1)

where *I_SERS_* and *I_REF_* are the intensities of the 4-MBA peak at 1076 cm^−1^ in the SERS spectrum and at 1084 cm^−1^ in the Raman spectrum, respectively. Similarly, *N_SERS_* and *N_REF_* are the number of 4-MBA molecules contributing to the SERS and the Raman signals [28]. We realized the measurements in a dry state and the estimated values were: *I_SERS_* = 68320 counts, *I_REF_* = 2720 counts, *N_SERS_* = 1.44 × 10^6^ mol and *N_REF_* = 2.2 × 10^11^ mol, therefore achieving an average EF for our ThMo structure [39,43] of 3.8 × 10^6^ that suited our purpose of microorganism detection. EF > 10^6^ are indeed adequate to reveal *Brucella* at the single-cell level in aqueous suspension, as reported by Rippa et al. [12].

For the specific capture of the pathogen, the 4-ATP modified sensor was further functionalized with the bacteriophage Tb (Podoviridae family) via diazotization. The diazotization, also known as azo coupling, is an electrophilic aromatic substitution between a nucleophilic arene and a diazonium cation (i.e., the electrophile) to generate an azocompound. In this case, the diazonium (4-ATP–N≡N+) is formed by the reaction of the 4-ATP primary amine with the nitrous acid. The following conjugation of Tb to the diazonium takes place through the phage histidine/tyrosine residues that can act as nucleophiles [42]. After the diazo bond formation, well-distinguishable vibrations [28,42] appeared in the SERS spectrum. In particular, the comparison between the 4-ATP SERS spectrum (green line, Figure 4a) and the other spectra reported in Figure 4a (diazonium spectrum, blue curve; covalently immobilized phage, black curve; captured *Brucella*, red curve) allowed us to clearly note the presence of a new peak at 1322 cm^−1^ related to the vibrational stretching of the diazo-bond (4-ATP–N=N–Tb) [39,42]. This peak at 1322 cm^−1^ represents a valuable SERS marker [28,42] and we used its area as reference. In particular, we calculated the area increase of this reference peak due to a binding event, as previously reported [28]. A remarkable 30-fold amplification of the reference peak after phage immobilization was estimated with respect to the diazonium (4-ATP–N≡N+) spectrum (Figure 4a).

At first, we detected the *B. abortus* presence in aqueous samples (10^5^ CFU/mL), noticing an amplification of 1.3-fold for the peak located at 1322 cm^−1^ (red line, Figure 4b and Table 1).

As previously reported in literature [44], the SERS is based on a very short-range phenomenon and it is possible to assess via SERS the complete Raman bands only for the chemical moieties closest to the gold nanosurface. For this reason, we were able to assess the diazonium (4-ATP–N≡N+) SERS spectra but the successive binding events slightly affected the achieved Raman band pattern [44,45]. Binding events occurring on the diazonium (4-ATP–N≡N+) layer were however detectable. Indeed, they resulted in changes in the signal enhancement, according to the literature [12,44]. The Raman enhancement was not exclusive for just a single peak of the spectrum (Table 1) [12,39,44], but for convenience we chose the peak at 1322 cm^−1^ (related to the vibrational stretching of the diazo-bond) as a reference to evaluate the signal increment consequent to the binding. To facilitate the spectra comparison, the SERS spectra were referenced to the zero level and were normalized by setting the maximum intensity of the *Brucella* spectrum at 1 (as in Figure 4b).

The performances of our sensor were then tested analyzing the presence of live *B. abortus* cells (10^5^ CFU/mL) in a food matrix, i.e., micro-filtrated milk (Figure 5).

We demonstrated that quasi-crystal patterned NCs functionalized with 4-ATP and Tb bacteriophage allowed the effective SERS detection of ≈ 10^4^ viable *Brucella abortus* cells from a reduced sample volume.

The presented SERS substrate had an EF of 3.8 × 10^6^, suitable to reveal the microorganism at single-cell level [12], and a 4-fold intensity enhancement of the marker peak at 1322 cm^−1^ (vibrational stretching of 4-ATP–N=N–Tb) was observed for the bacterium detection in milk. Such performance was due to both the covalent Tb attachment to the sensor surface via diazotisation and the specific phage recognition of the pathogen.

These results indicated, for the first time to our best knowledge, that SERS spectroscopy in combination with ThMo metastructures and a 4-ATP–N=N–Tb functionalisation can offer a reliable and easy alternative for in situ detection of *Brucella* also in a real matrix such as milk. Work is in progress to detect the pathogen in clinical matrices for diagnostic purposes.

## Figures and Tables

**Figure 1:**
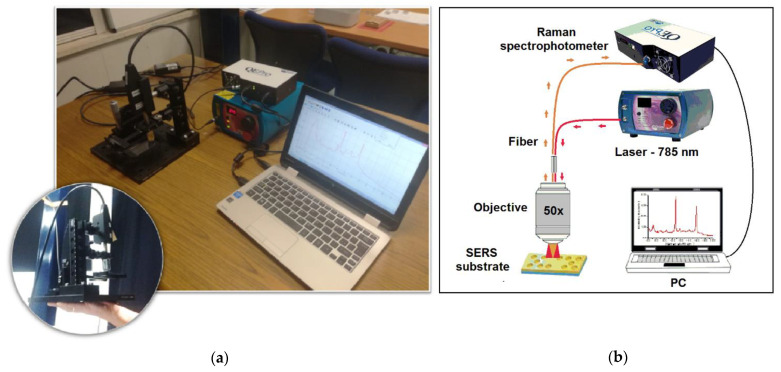
Home-made portable system for Surface Enhanced Raman Spectroscopy (SERS) analysis in situ: (**a**) Picture of the system, (**b**) schematic representation.

**Figure 2:**
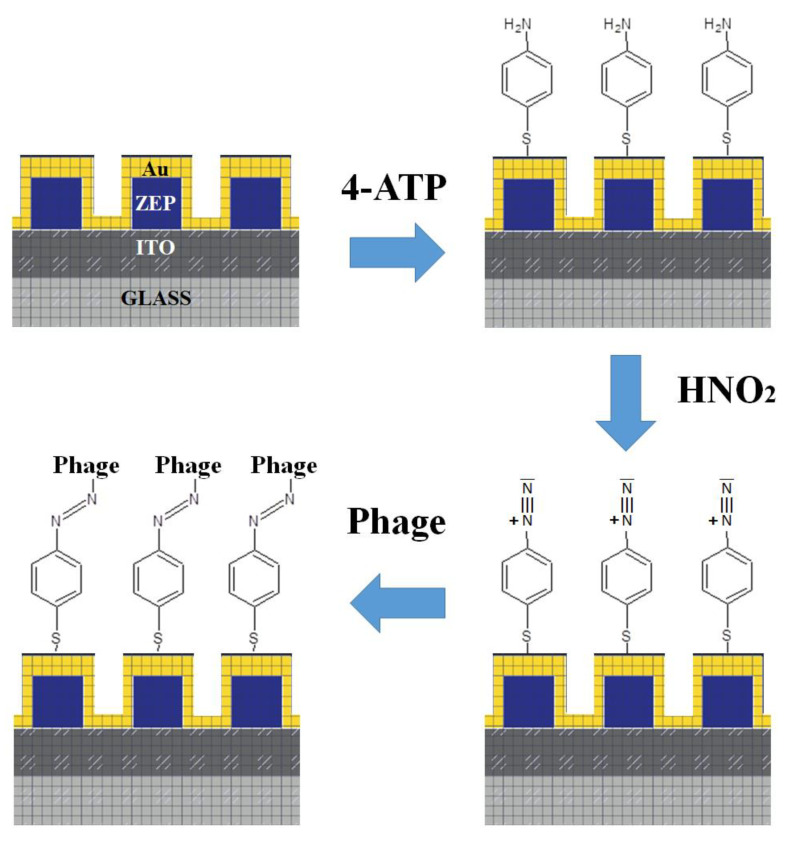
Schematic representation of the patterned NCs and the Au-surface functionalization.

**Figure 3:**
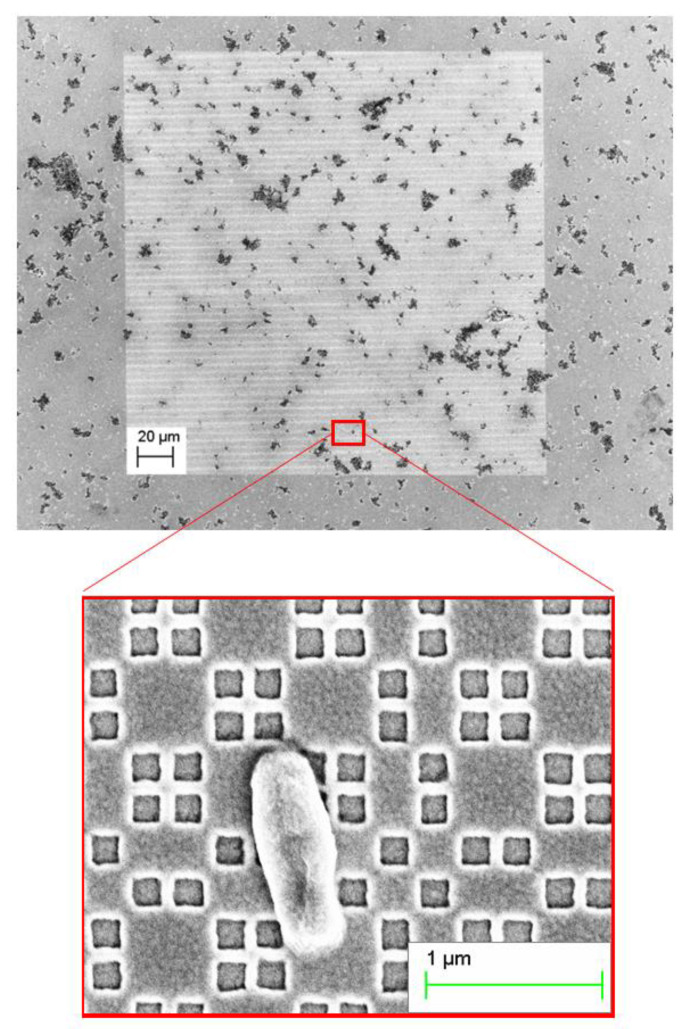
Morphological characterization of the nanostructures by SEM. Detailed image of the structure showing a cell of *Brucella abortus* is highlighted in red. Structure measures are distance of a = 50 nm, and side size of d = 185 nm with increasing edge-to-edge distances from 25 to 100 nm.

**Figure 4:**
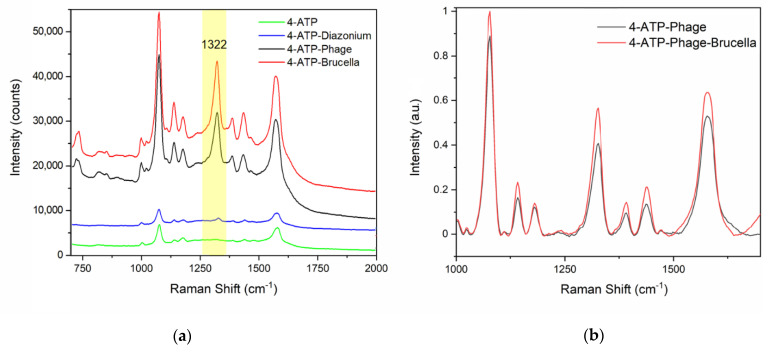
Comparison of SERS measurements performed on our functionalized nanostructures after binding in ddH_2_O. (**a**) Registered SERS spectra of the 4-ATP SAM (green line), the diazonium (4-ATP–N≡N+) (blue curve), the covalently immobilized phage (black curve), and the captured *Brucella* (red curve). (**b**) Magnification of the SERS spectra referenced to the zero level for the covalently immobilized phage (black curve) and the captured *Brucella* (red curve).

**Figure 5:**
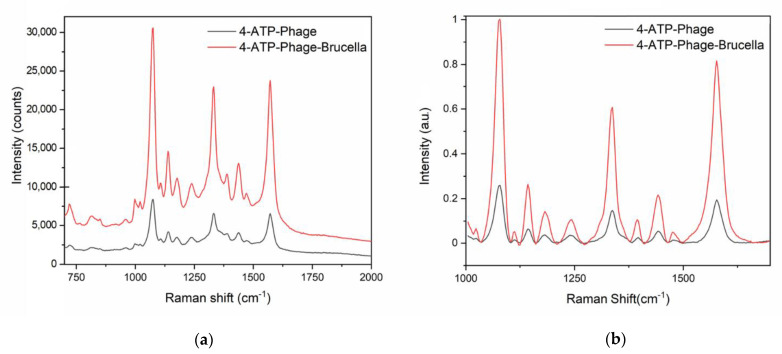
Comparison of SERS measurements performed on our functionalized nanostructures after binding in milk. (**a**) Registered SERS spectra of the covalently immobilized phage (black curve) and the captured *Brucella.* (**b**) Magnification of 40-min incubation of the Tb-sensor with milk free of pathogens (black line, Figure 5). Micro-filtrated milk was then inoculated with the live bacteria. The spiked sample (300 µL, total bacterial count of 3 × 10^4^ CFU) was dispersed onto the biosensor surface and incubated for 40 min. The 1322 cm^−1^ peak recorded for the contaminated sample (red line, Figure 5b) had a 4-fold larger area than the reference peak of the negative control spectrum (black line, Figure 5b and Table 1). These results proved the outstanding performances of our biosensor in real conditions, i.e., with a viable form of the pathogen and a complex food matrix. Interesting to note, the signal amplification achieved for *B. abortus* detection in milk was even higher than in distilled water at equal bacterial count. A plausible reason for this discrepancy could lie in the pH/ionic strength values of the two environments that differently influenced the phage binding.

**Table 1:** Vibrational assignment of Raman and SERS spectra.

Vibrational Assignment	4-ATP Raman (cm^−1^)	4-ATP SERS (cm^−1^)	Tb Phage (cm^−1^)	*Brucella* Enhancement in ddH_2_O ^1^	*Brucella* Enhancement in Milk ^1^
SC str + NH_2_ rock	1086 s	1076	1075 s	1.1	3.9
CH bend	1174 w	1177	1177 m	1.1	3.8
CN bend	1206 vw	-	-	-	-
CH str	1288 w	1308	-	-	-
NN str	-	-	1322 s	1.3	4
CC str + CH + rock + NH_2_rock	-	1391	1388 w	1.2	1.2
CC str + NH_2_ rock	-	1440	1436 m	1.5	0.8
CC str + CH bend	1491 w	1478	-	-	-
CC str + NH_2_ bend	1590 s	1581	1574 s	1.3	4.3
SH str	2555	-	-	-	-

^1^ The areas of the fitted peaks were used to calculate the enhancement of the signal as compared to the negative control (phage signal without the bacteria).

## Data Availability

The data presented in this study are available on request from the corresponding author.
